# Progress in Surgery of the Liver and Gall Bladder

**Published:** 1903-10-17

**Authors:** 


### Progress in Surgery of the Liver and Gall Bladder.

**Some Practical Points in the Anatomy of the Gall-bladder Region** are given by Brewer.[1] The important points are :—1. A knowledge of the chief nerve trunks of the abdominal wall. 2. The normal position, size, and shape of the gall-bladder and ducts, and of certain constant lymph nodes. 3. Certain variations from the normal in these structures. 4. Variations in the blood supply of the liver and gall-bladder. 5. The relation of the duodenal orifice at the common bile-duct. If an incision through the rectus muscle is employed, extending from a point 1 cm. below the eighth costal cartilage to 1 cm. above the umbilicus, only the ninth intercostal nerve will be divided. The mucous membrane of the cystic duct contains a series of folds or valves varying in number from four to twenty. These valves generally prevent the passage of the probe in either direction. The average length of the common duct is 7·5 cm., and its diameter 20 mm. The right or free extremity of the gastro-hepatic omentum frequently contains branches of the artery derived from other sources than the hepatic artery, and this should be remembered in tearing away the gastro-hepatic omentum.

Cholelithiasis.—Lartigan[2] says it has been shown that the phenomena of cholelithiasis are closely related with bacterial invasion of the gall-bladder. His own studies show that the growth of micro-organisms, such as the colon and typhoid bacilli, are but slightly inhibited by bile. It is probable that the portal route from the intestine is a common channel of infection of the gall-bladder in cases of cholelithiasis. The author lays great stress upon the stagnation of the bile as an important factor in the production of cholelithiasis. Zeller[3] considers it necessary to open the duodenum and pass a probe through the papilla if one fails to find the calculus by searching along the under surface of the liver and along the course of the ducts. He finds that the best incision in the bowel is exactly in the middle of the descending portion of the duodenum, and it should be made transversely to the long axis of the bowel. On opening the bowel one must look for the longitudinal fold, and one will then have no difficulty in recognising the papilla; but if one neglects to look for the plica one may mistake the papilla duodenalis minor, in which the accessory pancreatic duct ends, for the papilla of Vater. At times it will be found much easier to dislodge a calculus by passing a sound upwards than downwards from the gall-bladder. The suture of the bowel wall is much simpler than that of the gall-bladder. Mayo[4] urges early operation for gall-stones. In over 2,000 operations of this kind in the hands of six surgeons there was not a single instance of reformation of gall-stones. In this field of surgery delay breeds misfortune. By the "ideal" operation of cholecystendycis stones are apt to be overlooked. Cholecystotomy and drainage is the operation of choice. Drainage is continued until the bile is normal. Gall-bladders with thickened walls, and especially if the cystic duct has been obstructed, are liable to give trouble after cholecystotomy, and, if possible, the organ should be removed. If the liver-ducts are free from involvement, then, and then only, should the cystic duct be tied and the gall-bladder excised without drainage of the bile from the liver-ducts. Kehr says that the hepatic ducts require drainage in 37 per cent. of cases. In stones in the common duct drainage of the hepatic duct is essential. This usually has been done by suturing the common duct and draining through the gall-bladder by cholecystotomy. Radsiewsky[5] collected 56 cases in which the gall-bladder was either joined to the colon or small intestine, including the duodenum, and in not one single case was a definite cholangitis purulenta to be found *post mortem.* He therefore concludes that there is no danger of spread of infection upwards from the intestine. Mayo Robson[6] now always makes his incision over the middle of the right rectus in a line parallel with its fibres, which are then separated by the finger, the posterior sheath of the rectus and the peritoneum being divided together. Where the gall-bladder is distended, and there is no jaundice, a small incision of only 2 or 3 inches, may be required; but when it is necessary to explore either the hepatic, common, or deeper part of the cystic duct, instead of prolonging the incision downwards, as was formerly done, he now carries it upwards in the interval between the ensiform cartilage and the right costal margin as high as possible, thus exposing the upper surface of the liver very freely. It will be found that by lifting the lower border of the liver in bulk the whole of the gall-bladder and the cystic and common ducts are brought quite close to the surface; and as the gall-bladder is usually strong enough an assistant can take hold of it and by gentle traction can keep the parts well exposed, at the same time that by means of his left hand, with a flat sponge under it, he retracts the left side of the wound and the viscera, which would otherwise fall over the common duct and impede the view. A firm sandbag about 18 inches long by 6 inches wide and 3½ inches deep, covered with flannel and placed on the operating table at the liver level, will push the spine forwards, and with it the liver and bile-ducts, so that the common and hepatic ducts are brought several inches nearer the surface. By opening out the costal angle and tending to make the intestines slip down from the liver it acts like the Trendelenberg position in pelvic surgery.

[1] Med. Rec., New York, Dec. 27, 1902, p. 1033. [2] Ibid., p. 1034. [3] Brit. Med. Jour., Nov. 15, 1902, Epitome No. 297. [4] Med. Rec., New York, Feb. 21, 1903, p. 289. [5] Scottish Med. and Surg. Jour., Dec. 1902, p. 550. [6] Brit. Med. Jour., Jan. 24, 1903, p. 182.

*(To be continued.)*

